# The anti-inflammatory effect of LMWF5A and N-acetyl kynurenine on macrophages: Involvement of aryl hydrocarbon receptor in mechanism of action

**DOI:** 10.1016/j.bbrep.2018.06.006

**Published:** 2018-07-11

**Authors:** Leonard T. Rael, Raphael Bar-Or, Kaysie L. Banton, Charles W. Mains, Michael Roshon, Allen H. Tanner, Mark J. Lieser, David L. Acuna, David Bar-Or

**Affiliations:** a Swedish Medical Center, Englewood, CO 80113, USA; b St. Anthony Hospital, Lakewood, CO 80228, USA; c Medical City Plano, Plano, TX 75075, USA; d Penrose Hospital, Colorado Springs, CO 80907, USA; e Research Medical Center, Kansas City, MO 64132, USA; f Wesley Medical Center, Wichita, KS 67214, USA; g Ampio Pharmaceuticals, Inc., Englewood, CO 80112, USA; h Rocky Vista University, Parker, CO 80134, USA

**Keywords:** NAT, N-acetyl tryptophan, KYN, kynurenine, NAK, N-acetyl kynurenine, HSA, human serum albumin, LMWF5A, low molecular weight fraction of 5% albumin, ESI+, electrospray positive ionization, LCMS, liquid chromatography-mass spectrometry, AhR, aryl hydrocarbon receptor, Tryptophan, Inflammation, Macrophages, Aryl hydrocarbon receptor, Kynurenine, Human serum albumin

## Abstract

After a traumatic insult, macrophages can become activated leading to general inflammation at the site of injury. Activated macrophages are partially regulated by the aryl hydrocarbon receptor (AhR) which when activated suppresses inflammation by limiting the secretion of pro-inflammatory cytokines and promoting the over expression of immuno-modulatory mediators. This study aims to determine whether the low molecular weight fraction of 5% human serum albumin (LMWF5A) and N-acetyl kynurenine (NAK), an N-acetyl tryptophan (NAT) breakdown product in LMWF5A, can regulate inflammation by inhibiting macrophage activation through the AhR since kynurenine is a known AhR agonist. Using LCMS, we demonstrate that NAT is non-enzymatically degraded during accelerated aging of LMWF5A with high heat accelerating degradation. More importantly, NAK is a major degradation product found in LMWF5A. THP-1 monocytes were differentiated into macrophages using phorbol 12-myristate 13-acetate (PMA) and pre-treated with 2-fold dilutions of LMWF5A or synthetic NAK with or without an AhR antagonist (CH223191) prior to overnight stimulation with lipopolysaccharide (LPS). Treatment with LMWF5A caused a 50–70% decrease in IL-6 release throughout the dilution series. A dose-response inhibition of IL-6 release was observed for NAK with maximal inhibition (50%) seen at the highest NAK concentration. Finally, an AhR antagonist partially blocked the anti-inflammatory effect of LMWF5A while completely blocking the effect of NAK. A similar inhibitory effect was observed for CXCL-10, but the AhR antagonist was not effective suggesting additional mechanisms for CXCL-10 release. These preliminary findings suggest that LMWF5A and NAK partially promote the suppression of activated macrophages via the AhR receptor. Therefore, LMWF5A, which contains NAK, is potentially a useful therapeutic in medical conditions where inflammation is prevalent such as trauma, sepsis, and wound healing.

## Highlights

• LMWF5A contains degradation products of N-acetyl tryptophan (NAT).
• A major degradation product of NAT in LMWF5A is N-acetyl kynurenine (NAK).
• LMWF5A and NAK decrease IL-6 and CXCL-10 release from activated macrophages.
• AhR is partially involved in the mechanism of action for LMWF5A and NAK.
• The anti-inflammatory properties of LMWF5A are also AhR-independent.

## 1 Introduction

Human serum albumin (HSA) is clinically used for the treatment of shock, acute restoration of blood volume, and hypoalbuminemia. In recently completed clinical studies, the low molecular weight fraction of 5% HSA (LMWF5A) has demonstrated efficacy in the treatment of knee osteoarthritis (OA) [1], [2]. These improvements in knee OA symptoms include decreases in pain and increases in overall function. Recently published findings have documented biochemical and cellular events that assist in elucidating the anti-inflammatory effects of LMWF5A using various in vitro assays. In a cell culture model using bone marrow-derived mesenchymal stem cells, LMWF5A was shown to prime these cells for both mobilization and chondrogenic differentiation [3]. LMWF5A was also shown to significantly decrease TNFα release in LPS-stimulated human peripheral blood mononuclear cells (PBMC) [4]. Finally, the effect of LMWF5A on important mediators of pain and healing in knee OA such as prostaglandins has been suggested [5]. Indeed, LMWF5A has been shown to increase the release of prostaglandins involved in the resolution of inflammation in a PBMC model [6]. All of these anti-inflammatory and pro-healing observations occur far downstream from a possible membrane-initiating effect of LMWF5A. Therefore, the search for potential membrane targets of LMWF5A is warranted.

Various components of LMWF5A possess anti-inflammatory properties that might be relevant to nociception and initiation of healing. For example, the cyclic compound derived from the N-terminus of HSA, aspartate-alanine diketopiperazine (DA-DKP), is found in LMWF5A in micromolar concentrations that are high enough to decrease pro-inflammatory cytokine release from PBMC and T-cell lines following stimulation [7], [8]. An excipient added to 5% HSA solutions, N-acetyl tryptophan (NAT), is known to have immuno-modulatory properties via inhibition of neurokinin 1 receptor (NK1R) thereby regulating important pro-inflammatory signals in immune cells [9], [10]. NAT degradation products have also been identified in commercial HSA solutions with unknown biological activity [11]. Finally, various non-HSA derived peptides that co-elute with the HSA fraction have been characterized in commercial HSA solutions [12], [13]. As a result, it is possible that any combination of known and unknown small molecular weight components of commercial HSA solutions contributes to the anti-inflammatory properties of LMWF5A.

Because it is present in large concentrations (4 mM) in 5% commercial HSA solutions, an obvious active ingredient candidate in LMWF5A is NAT. Besides the already discussed inhibitory effect on NK1R, it is possible that metabolites of NAT also have biological effects. This hypothesis is based on the documented biological activities of metabolites of the amino acid tryptophan. The oxidation of tryptophan is a complex metabolic pathway that results in the production of kynurenine (KYN) and associated biologically active molecules [14]. Additionally, tryptophan metabolites have certain redox properties making them important in aging and neurodegenerative processes [15]. Therefore, the purpose of this study is to characterize LMWF5A using LCMS-MS to identify non-enzymatic breakdown products of NAT. Additionally, the anti-inflammatory effect of LMWF5A and a synthetic NAT breakdown product, N-acetyl kynurenine (NAK), will be assessed using differentiated THP-1 macrophages stimulated with LPS with special emphasis on the involvement of the aryl hydrocarbon receptor (AhR) in the mode of action of LMWF5A.

## 2 Materials and methods

### 2.1 Materials

5% human serum albumin (HSA) (Octapharma, Hoboken, NJ) was used for the production of LMWF5A. LCMS solvents were purchased from Fisher Scientific (Pittsburgh, PA). 0.9% (w/v) sodium chloride (10 ml saline injection syringe flush, USP) was obtained from Excelsior Medical (Neptune, NJ, USA). All cell culture reagents were obtained from Thermo Fisher Scientific (Waltham, MA). A 1 mg/ml PMA stock was made in DMSO, and a 1 mg/ml LPS (O55:B5) stock was made in non-supplemented RPMI 1640 media. N-acetyl kynurenine (NAK) was synthesized by IsoSciences (King of Prussia, PA). All other reagents were obtained from Sigma (St. Louis, MO) unless otherwise stated.

### 2.2 Collection of LMWF5A

LMWF5A was isolated by Ampio Pharmaceuticals, Inc. (Englewood, CO, USA) using a tangential flow filtration (TFF) process with a 5 kDa MWCO PVDF filter membrane (Sartorius Stedim Biotech GmbH, Germany). In accordance with cGMP guidelines, the isolation process involved the removal of the > 5 kDa component (primarily HSA) and the aseptic filling of sterile glass vials with 4.2 ml LMWF5A. Each vial was sealed with a rubber stopper and a proper metal closure. The vials were stored in the dark at ambient temperature.

### 2.3 LCMS Analysis of LMWF5A

LMWF5A was injected on an Acquity UPLC BEH C18 column (Waters, Milford, MA, USA) connected to an Acquity H-Class liquid chromatography system (Waters, Milford, MA, USA) and Xevo G2S tandem mass spectrometer (Waters, Milford, MA, USA). Starting mobile phase conditions consisted of 99% HPLC-grade water with 0.1% TFA (Solvent A) and 1% acetonitrile with 0.1% TFA (Solvent B) at a flow rate of 0.5 ml/min. The gradient was adjusted to 40% Solvent A and 60% Solvent B during the 25 min run. A 5 min equilibration was included to return to starting conditions. MS survey conditions consisted of capillary (2.5 kV), sampling cone (30 V), source temperature (110 °C), desolvation temperature (500 °C), cone gas (150 L/h), desolvation gas (850 L/h) and collision energy (6 V). Accurate mass determination was accomplished using leucine enkephalin. MS-MS was performed using the same conditions as the LCMS settings above except the collision energy was set at 15 V.

### 2.4 THP-1 cell differentiation and dosing

All cell cultures were incubated at 37 °C and 5% CO_2_. Suspensions of human THP-1 monocytes (ATCC, Manassas, VA) were initially cultured in 75 cm^2^ flasks at 2–3 × 10^5^/ml in RPMI 1640 media supplemented with 10% fetal calf serum (FCS) and 1% penicillin/ streptomycin (Pen/Strep). Cells were counted with trypan blue, and 1 × 10^5^cells were added to each well of a 96-well flat bottom plate. 50 ng/ml (final) phorbol 12-myristate 13 acetate (PMA) was added to all wells.

Dosing solutions consisted of 2 × dilutions of LMWF5A or 200 µM NAK in saline. Since the starting solutions are saline-based, the dosing solutions were diluted 1:1 with RPMI 1640 supplemented with 20% FCS, 2% Pen/Strep, 1% L-glutamine, 1% sodium bicarbonate, 1% sodium pyruvate, and 1% non-essential amino acids (NEAA). After 3 days of differentiation with PMA, the media was aspirated from each well. To triplicate wells, 200 µl of the appropriate dosing solution was added with or without the AhR antagonist (CH223191, final concentration of 10 µM) and incubated for 1 h. 20 µl of 1.1 µg/ml LPS (final = 100 ng/ml) was added to all wells and incubated overnight.

### 2.5 qPCR Toll-like receptor signaling pathway array

In 25 cm^2^ flasks, 3 × 10^5^/cm^2^ THP-1 cells were plated and differentiated as described above in Section 2.4 scaling up to 10 ml total volume. Flasks were dosed and stimulated scaling up to 8.8 ml total volume (8 ml 2 × diluted LMWF5A + 0.8 ml 1.1 µg/ml LPS). After the overnight incubation, media was aspirated from all flasks. RNA was isolated from each flask using RNeasy Plus Mini Kit and QIAshredder spin columns (Qiagen, Hilden, Germany). 0.5 µg of total RNA was then reverse transcribed into cDNA with QuantiTect kit (Qiagen, Hilden, Germany). 4.25 ng cDNA was added to each well of a 96-well RT^2^ Profiler PCR Array for the Human Toll-Like Receptor Signaling Pathway (Qiagen, Hilden, Germany). Real time (RT) PCR was performed using a 480 Lightcycler (Roche Diagnostics, Indianapolis, IN). Relative gene expression was calculated using the comparative threshold cycle (ΔΔC_T_) method versus LPS only with normalization to internal controls (actin, β − 2-microglobulin, and GAPDH).

### 2.6 ELISA for Biomarkers of Inflammation

After an overnight LPS stimulation, supernatants were collected and analyzed by ELISA for IL-6, IL-10, IL-12, and CXCL-10 (Thermo Fisher Scientific, Waltham, MA).

### 2.7 Data analysis

For the qPCR data, fold-change (2^(-ΔΔC_T_)) is the normalized gene expression (2^(-ΔC_T_)) in the Test Sample divided by the normalized gene expression (2^(-ΔC_T_)) in the Control Sample. Fold-regulation represents fold-change results in a biologically meaningful way. A Fold-change value > 1 indicates a positive- or an up-regulation, and the fold-regulation is equal to the fold-change. Fold-change values less than one indicate a negative or down-regulation, and the fold-regulation is the negative inverse of the fold-change.

A Mann-Whitney U non-parametric test was applied to all data sets with statistical significance accepted at α < 0.05.

## 3 Results

### 3.1 LCMS analysis of LMWF5A

**Fig. 1:**
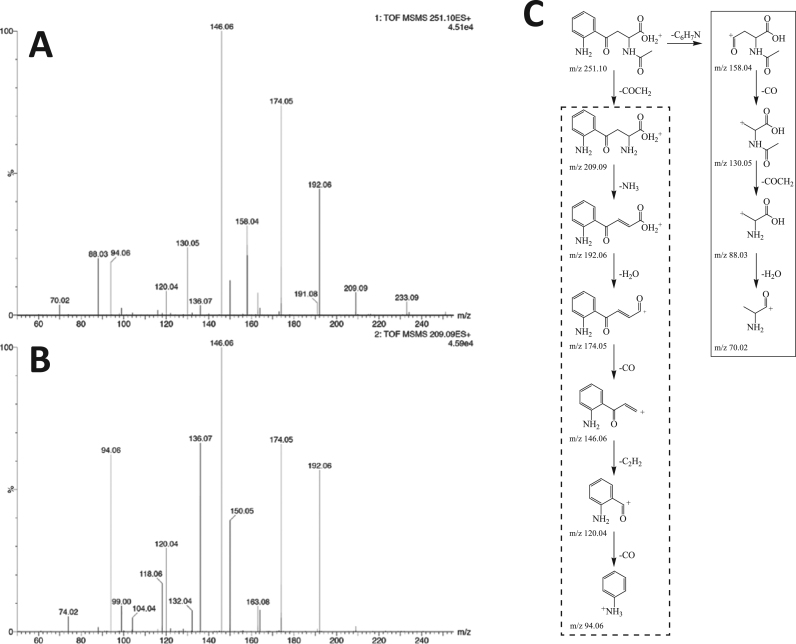
LCMS-MS spectra of NAK ([M+] = 251.10) (A) identified in LMWF5A and synthetic KYN ([M+] = 209.09) in saline (B). Structural similarities include mass fragments at *m/z* 192.06, 174.05, 146.06, 120.04, and 94.06. Structural differences include mass fragments at *m/z* 158.04, 130.05, 88.03, and 70.02 seen in the NAK spectrum only. (C) Proposed fragment structures for the observed product ions using the LCMS-MS conditions listed in the Materials and Methods section. The structural similarities of NAK ([M+] = 251.10) and KYN ([M+] = 209.09) include mass fragments at *m/z* 192.06, 174.05, 146.06, 120.04, and 94.06 (dotted box). The structural differences of NAK and KYN include mass fragments at *m/z* 158.04, 130.05, 88.03, and 70.02 (solid box). A synthetic standard of NAK was analyzed by LCMS and shown to be structurally identical to [M+ ] = 251.10 identified in LMWF5A (data not shown).

A peak corresponding to the molecular mass of NAK ([M+] = 251.10) was identified in LMWF5A. MS-MS was performed to obtain structural information (Fig. 1A). To determine if this molecule is structurally related to kynurenine (KYN), synthetic KYN was also subjected to the same MS-MS conditions at [M+ ] = 209.09 (Fig. 1B). The two spectra show strong similarities in that major fragment peaks are observed at *m/z* of 192.06, 174.05, 146.06, 120.04, and 94.06. These fragments are presented within the dashed box in Fig. 1C. Also, the mass of the parent peak of KYN (*m/z* 209.09) was observed in Fig. 1A indicating that the acetyl group is cleaved from NAK under these MS-MS conditions.

Significant differences between the two MS-MS spectra at *m/z* of 158.04, 130.05, 88.03, and 70.02 were observed for NAK (Fig. 1A). For the mass fragments at *m/z* of 158.04 and 130.05, these correspond to fragments that still contain the N-acetyl group thereby strongly suggesting that the parent compound is NAK (Fig. 1C – solid box). The mass fragment at *m/z* of 88.03 is also present in the KYN spectra at much lower intensity. This fragment does not contain the N-acetyl group, but it is more intense in the NAK spectra likely due to the specific MS conditions. The same argument can be made for the mass fragment at *m/z* 70.02. Additionally, a synthetic standard of NAK was analyzed by LCMS and shown to be structurally identical to [M+ ] = 251.10 identified in LMWF5A (data not shown). Finally, an average concentration of 200 µM NAK was quantified in multiple lots of LMWF5A (data not shown) and therefore used in determining the proper NAK concentration for dosing differentiated THP-1 macrophages.

### 3.2 Effect of LMWF5A on Toll-like receptor signaling pathway

**Fig. 2:**
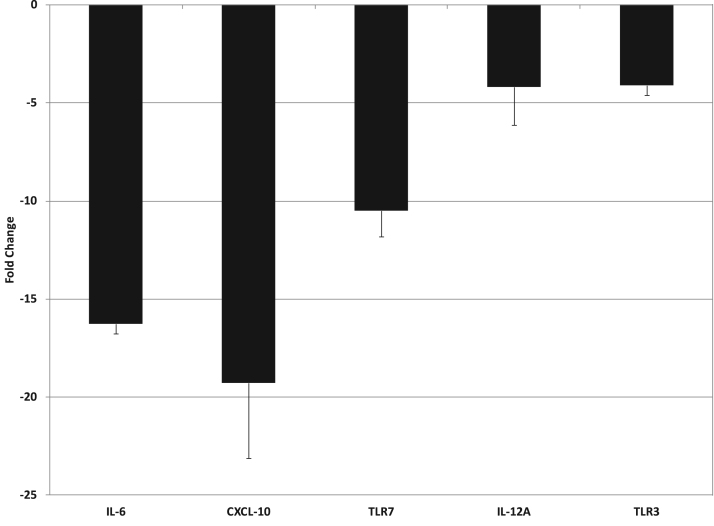
LMWF5A decreases mRNA for IL-6, CXCL-10, IL-12, and specific Toll-like receptors (TLR3 & TLR7) in PMA-differentiated THP-1 cells stimulated with LPS (100 ng/ml). Total RNA was isolated from THP-1 cells treated with saline or LMWF5A (N = 3). RT-qPCR was performed using a profiler array kit specific for the Toll-like receptor signaling pathway (Qiagen, Hilden, Germany). Using the ΔΔC_T_ method, relative fold changes in the LMWF5A-treated differentiated THP-1 cells were quantified versus saline-treated differentiated THP-1 cells. All RT-qPCR data was normalized to internal housekeeping genes included in 96-well array. Data is presented as fold change ± SD. All presented data is significant (α < 0.05) versus saline control.

Human THP-1 monocytes were differentiated into macrophages by treating with PMA. Macrophages were then stimulated overnight with LPS in the presence of saline or 2 × diluted LMWF5A (N = 3). RNA was isolated from each flask and then reversed transcribed into cDNA prior to addition to a 96-well RT-qPCR array specific for the Toll-like receptor signaling pathway. A minimum threshold of a 4-fold change was applied to the PCR data. Transcription of mRNA for IL-6 and CXCL-10 significantly decreased 16.3-fold ( ± 0.5) and 19.3-fold ( ± 3.8), respectively (Fig. 2). IL-12A mRNA also significantly decreased 4.2-fold ( ± 1.9) while mRNA for the Toll-like receptors TLR3 and TLR7 significantly decreased 4.1-fold ( ± 0.5) and 10.5-fold ( ± 1.3), respectively (Fig. 2). No significant changes were observed in unstimulated macrophages regardless of treatment type (data not shown).

### 3.3 Effect of LMWF5A on released markers of inflammation

Differentiated THP-1 macrophages were stimulated overnight in triplicate wells with 100 ng/ml LPS in the presence of saline or a manufactured lot of LMWF5A (3 lots total). Supernatants were collected and tested on 3 separate experiments for IL-6, IL-10, IL-12, and CXCL-10 release by commercially available ELISA kits. IL-6 release significantly decreased by 49% in LPS-stimulated differentiated THP-1 cells treated with LMWF5A (Table). An anti-inflammatory cytokine (IL-10) significantly increased by 2.3-fold while IL-12 release significantly decreased by 45% when treated with LMWF5A (Table). Finally, the release of the pro-inflammatory chemokine CXCL-10 demonstrated a statistically significant 62% decrease after LMWF5A treatment (Table).

**Table:** Effect of LMWF5A on Markers of M1 (IL-6, IL-12, CXCL-10) or M2 (IL-10) Macrophages.

Treatment	IL-6 pg/ml (SD)	IL-10 pg/ml (SD)	IL-12 pg/ml (SD)	CXCL-10 pg/ml (SD)
Saline+LPS	1032 (216)	9.5 (1.4)	549 (103)	24,107 (3613)
LMWF5A+LPS	530 (53)*	22.3 (2.0)*	303 (44)*	9072 (1711)*

Significance (α = 0.05) versus Saline+LPS is indicated with an asterisk (*).

**Fig. 3:**
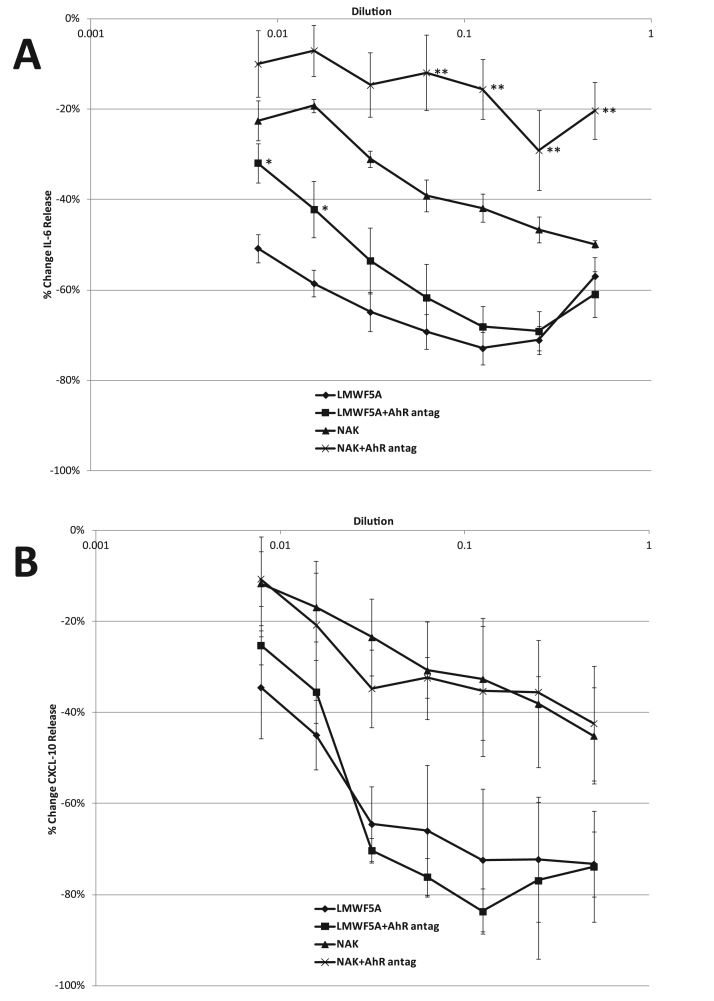
LMWF5A and NAK decrease the release of IL-6 (A) and CXCL-10 (B) in PMA-differentiated THP-1 cells stimulated with LPS (100 ng/ml). Data is presented as % change in release versus saline/LPS ± SD (N = 3). Statistical significance (α < 0.05) is indicated for LMWF5A with AhR antagonist versus LMWF5A only (*) or for NAK with AhR antagonist versus NAK only (**).

Differentiated THP-1 macrophages were stimulated overnight in triplicate wells with LPS in the presence of 1:2 serial dilutions of LMWF5A or NAK (starting [NAK] = 200 µM) with or without the AhR antagonist (10 µM CH223191). Supernatants were tested on 3 separate experiments for IL-6 and CXCL-10 release using commercially available ELISA kits. IL-6 release significantly decreased (p < 0.05) by 50–70% in LPS-stimulated differentiated THP-1 macrophages treated with serial dilutions of LMWF5A versus saline (Fig. 3A). Serial dilutions of NAK caused a dose-response significant decrease (p < 0.05) in IL-6 release with a maximum inhibition of 50% observed at the highest NAK concentration (Fig. 3A). The AhR antagonist partially blocked the IL-6 effect at higher dilutions of LMWF5A while significantly blocking (p < 0.05) the effect of NAK (Fig. 3A). The release of the chemokine CXCL-10 was also significantly inhibited (p < 0.05) by LMWF5A and NAK versus saline (Fig. 3B). However, the AhR antagonist had no effect on this inhibition. No significant changes were observed in IL-6 or CXCL-10 release from unstimulated macrophages regardless of treatment type (data not shown).

### 3.4 Additional breakdown products of NAT in LMWF5A

**Fig. 4:**
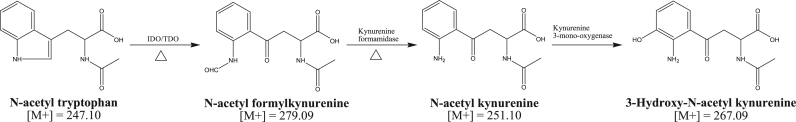
Proposed non-enzymatic breakdown of NAT in LMWF5A. Under thermal forced degradation conditions (Δ) of LMWF5A, the peak corresponding to the molecular weight of NAK ([M+] = 251.10) significantly increases. Also, [M+ ] = 279.09 increases with thermal forced degradation conditions (80 °C, 1 week) in LMWF5A suggesting the increased production of the precursor (N-acetyl formylkynurenine) to NAK. Finally, the mass ([M+] = 267.09) corresponding to a product (3-hydroxy-N-acetyl kynurenine) of NAK metabolism is present in LMWF5A but did not increase with heat indicating that an enzyme (kynurenine 3-mono-oxygenase) is necessary. The other enzymes involved in the metabolism of NAT are also included (IDO/TDO and kynurenine formamidase).

In addition to NAK, other significant NAT breakdown components of LMWF5A were identified by LCMS. These observed peaks might correspond to N-acetyl formylkynurenine (*m/z* 279.09) and 3-hydroxy-N-acetyl kynurenine (*m/z* 267.09) (Fig. 4). Accelerated aging increases the production of NAK in LMWF5A. The mass (*m/z* 279.09) corresponding to N-acetyl formylkynurenine, the precursor to NAK, increases by 20–25% with heat (80 °C, 1 week) indicating that formation of this degradation product of NAT can be achieved non-enzymatically. The mass (*m/z* 267.09) corresponding to 3-hydroxy-N-acetyl kynurenine, a product of NAK metabolism, is present in LMWF5A (Fig. 4). However, this mass does not increase with heat indicating that the formation of this degradation product is probably only achieved with an enzyme (kynurenine 3-mono-oxygenase, KMO).

## 4 Discussion

NAT is added at a concentration of 4 mM to 5% commercial solutions of HSA for the purpose of stabilizing the protein during the pasteurization process [16]. It has been postulated that NAT provides a protective effect on the free sulfhydryl group (Cys-34) of HSA thereby diminishing protein oxidation [17]. As a result of this anti-oxidant effect, the oxidative breakdown of NAT in commercial solutions of HSA has been described [18]. In this study, the low molecular weight fraction of 5% HSA (LMWF5A) was shown to contain breakdown products of NAT. This is in agreement with a previous study that identified and characterized NAT degradation products in HSA solutions using LCMS methodology [11]. However, to our knowledge, this is the first study to specifically describe the presence of NAK in a solution derived from commercially available HSA.

It is well established that the amino acid tryptophan is susceptible to degradation when exposed to UV light. UV exposure of tryptophan solutions resulted in significant photodegradation with a gradual color change and generation of reactive oxygen species [19]. These degradation products that caused the color change after UV exposure were attributed to major tryptophan oxidation products such as KYN, N-formylkynurenine, and hydroxytryptophan [20]. Although a similar color change is observed in LMWF5A over time, it cannot be attributed to exposure to UV rays since the solution is kept in the dark during storage. Protecting LMWF5A from light exposure still results in the production of NAT breakdown products suggesting a gradual, non-enzymatic process. This non-enzymatic process is enhanced during thermally forced degradation conditions (80 °C, 1 week) resulting in an increase in NAK and N-acetyl formylkynurenine formation in LMWF5A. Finally, an NAT-only solution heated at 80 °C also tested positive for kynurenine-like compounds (data not shown). This agrees with a study that observed no oxidation of a tryptophan solution heated at high temperature in the absence of oxygen but demonstrated first order degradation kinetics when the thermal treatment was conducted in the presence of oxygen [21].

It has been suggested that tryptophan metabolites act via the aryl hydrocarbon receptor (AhR) which upon activation increases the ratio between regulatory T cells (T_reg_) and T helper 17 cells (Th17) thereby modulating the immune response [22]. Not surprisingly, the tryptophan metabolites KYN and kynurenic acid (KA) are potent AhR agonists [23]. KYN and KA were included in this study at a concentration of 250 µM and inhibited CXCL-10 release by ~30% (data not shown). This interaction between kynurenine-like molecules and AhR is capable of generating immuno-suppressive T cells [24]. Finally, various studies have suggested that AhR activation regulates immune responses via interaction with key components of nuclear factor-κB (NF-κB), an important promoter of inflammation [25], [26]. In this study, we demonstrate that an AhR antagonist partially blocks the anti-inflammatory activity of LMWF5A on IL-6 release in LPS-stimulated macrophages. Additionally, NAK is an active component of LMWF5A since this compound also significantly inhibits IL-6 release. However, NAK activity is completely inhibited by the AhR antagonist suggesting that NAK is also an AhR agonist. Based on these preliminary findings, activation of the AhR pathway partially explains the mechanism of action of LMWF5A, but blockade of AhR has no effect on LMWF5A and inhibition of CXCL-10 release.

Macrophages are phagocytes found in essentially all tissues where a primary function is to engulf potential pathogens. They are involved in both innate immunity (nonspecific defense) and adaptive immunity (memory for subsequent insults). A primary function of innate immunity is the recruitment of other immune cells thereby leading to adaptive immunity. Macrophages recruit lymphocytes via the production and release of various chemical factors. Initially, this macrophage burst leads to inflammation characterized by pain, swelling, and fever. However, this process is important in combating the spread of infection thereby promoting healing and pathogen clearance. This immuno-regulatory property is partially due to the presence of moderate to high expression levels of AhR in macrophages [27]. Activation of the AhR pathway affects IL-6 transcription via inhibition of promoter activity [28] and also decreases IL-6 production through suppression of histamine production in macrophages stimulated with LPS [29]. No association was found between CXCL-10 and AhR after performing an extensive literature search. This further supports our findings that LMWF5A through NAK inhibits IL-6 release via the AhR but inhibits CXCL-10 release by an AhR-independent mechanism. Also, for IL-6 release, blockade of the AhR is only effective at low concentrations of LMWF5A suggesting that other anti-inflammatory components of LMWF5A or possibly NAT metabolism within the cell (i.e. conversion to NAK or other kynurenine-like molecules) saturate the AhR and overcome any AhR antagonism.

There are two main macrophage phenotypes: M1 or classically activated macrophages, and M2 or alternatively activated macrophages. M1 macrophages are described as “killer” macrophages, activated by LPS, secrete high levels of pro-inflammatory cytokines/chemokines (IL6, IL-12, CXCL-10), and low levels of IL-10 [30]. M2 macrophages and M2-like subtypes are seen as “repair” macrophages due to enhanced wound healing and tissue repair and can inhibit enhanced immune responses thereby decreasing inflammation via the release of high levels of IL-10 [30]. Of note, release of specific markers (IL-6, IL-12, and CXCL-10) of M1 macrophage activation was significantly decreased after treatment with LMWF5A and LPS stimulation. This is in agreement with the decrease in the transcription of these M1 activation markers obtained by RT-qPCR. Treatment with LMWF5A also significantly increased the release of a marker (IL-10) of M2 macrophage activation. These findings suggest that LMWF5A not only inhibits M1 activation but potentially favors M2 activation. This partially explains the in vivo mechanism of action for LMWF5A since inhibition of M1 macrophages and potential activation of an anti-inflammatory macrophage phenotype (M2) might lead to pain relief, wound healing, and tissue remodeling.

In conclusion, breakdown products of NAT were detected in LMWF5A with NAK being the dominant species. LMWF5A and NAK inhibit IL-6 and CXCL-10 release from activated macrophages via an AhR-dependent and -independent pathway, respectively. These preliminary findings partially explain the mechanism of action of LMWF5A in treating chronic conditions characterized by inflammation. Future studies should focus on downstream targets of AhR such as the AhR nuclear translocator (ARNT) and target genes such as CYP1A1/1B1 to fully elucidate the role of AhR in the overall anti-inflammatory effect of LMWF5A.

## Conflict of interest

L.T.R. is a shareholder, owns stock options, and uncompensated intellectual property rights at Ampio Pharmaceuticals. R.B.-O. is a compensated employee, shareholder, and owns stock options at Ampio Pharmaceuticals. D.B.-O. is a compensated employee, shareholder, owns stock options, and uncompensated intellectual property rights at Ampio Pharmaceuticals. All other authors have nothing to disclose.
